# Mathematics anxiety in children with developmental dyscalculia

**DOI:** 10.1186/1744-9081-6-46

**Published:** 2010-07-15

**Authors:** Orly Rubinsten, Rosemary Tannock

**Affiliations:** 1 Edmond J. Safra Brain Research Center for the Study of Learning Disabilities, Department of Learning Disabilities, University of Haifa, Israel; 2 Neuroscience and Mental Health Research Program, Hospital for Sick Children, Toronto, Ontario, Canada; 3 Human Development and Applied Psychology, Ontario Institute for Studies in Education, University of Toronto

## Abstract

**Background:**

Math anxiety, defined as a negative affective response to mathematics, is known to have deleterious effects on math performance in the general population. However, the assumption that math anxiety is directly related to math performance, has not yet been validated. Thus, our primary objective was to investigate the effects of math anxiety on numerical processing in children with specific deficits in the acquisition of math skills (Developmental Dyscalculia; DD) by using a novel affective priming task as an indirect measure.

**Methods:**

Participants (12 children with DD and 11 typically-developing peers) completed a novel priming task in which an arithmetic equation was preceded by one of four types of priming words (positive, neutral, negative or related to mathematics). Children were required to indicate whether the equation (simple math facts based on addition, subtraction, multiplication or division) was true or false. Typically, people respond to target stimuli more quickly after presentation of an affectively-related prime than after one that is unrelated affectively.

**Result:**

Participants with DD responded faster to targets that were preceded by both negative primes and math-related primes. A reversed pattern was present in the control group.

**Conclusion:**

These results reveal a direct link between emotions, arithmetic and low achievement in math. It is also suggested that arithmetic-affective priming might be used as an indirect measure of math anxiety.

## Background

In today's high-tech, increasingly connected world, it is vital that young children build confidence in their ability to do mathematics, as deficiencies in this area can be a major impediment to many facets of life. For example, mathematical impairments have been shown to be functionally significant for health numeracy [1,2], constraining informed patient choice and limiting access to available treatments. Moreover, math impairments have a negative influence on full-time employment in adulthood [3].

Mathematics anxiety [4] (henceforth, referred to as 'math anxiety'), is a negative reaction to math associated with negative emotions. Specifically, math anxiety is a state of discomfort occurring in response to situations involving mathematics tasks that are perceived as threatening to self-esteem [5]. It was claimed [6] that math anxiety manifests itself as an unpleasant emotional response to math. In this vein, Beilock and colleagues [7] raised an implicit argument that math anxiety is associated with negative attitude towards math [see also [8]]. Others [9] defined math anxiety as a feeling of tension, helplessness, mental disorganization and dread produced when one is required to manipulate numbers or to solve mathematical problems. Indeed, the everyday intuition that math is stressful has formal backing from the field of experimental psychology. For example, math is used as an experimental stressor in studies that look at cardiovascular activity [e.g., [10]].

Several studies have found that math anxiety and math achievement are negatively correlated [e.g., [6,8,11]]. It was found [11] for example, that across junior and senior high school, initial low math achievement is significantly related to later high math anxiety, but initial high math anxiety seems not to be strongly linked to later low math achievement. In primary school children [8] however, there was no clear developmental increase in the relationship of math anxiety and calculation abilities. In a later publication [12] a structural equation modeling found no effect of math anxiety on calculation ability. In the current study, our premise is that in Developmental Dyscalculia (DD; a deficit in processing numerical information) poor initial math abilities may precede and give rise to math anxiety, creating a vicious cycle.

In the present study, we show that math anxiety (1) is distinguishable from other types of anxiety symptoms (i.e., math anxiety can exist in the absence of more general anxiety traits); and (2) has a direct and deleterious effect on underlying cognitive processes as the individual performs a math task. We also show that this is especially true for individuals with DD. Prior to outlining our hypotheses and describing our study, we first highlight important developments in the understanding of math anxiety and dyscalculia.

## Math anxiety

Although not clearly discussed or scientifically studied, math anxiety is thought as specific to math context and therefore distinct and occurring in the absence of generalized anxiety [e.g., see [13] who extended Eysenck & Calvo's [14] predictions about generalized anxiety and working memory to math anxiety]. Accordingly, math anxiety seems to be a very prevalent phenomenon approaching, for example, 4% of high school students [15].

Reasons for math anxiety are usually classified as environmental, personal, or cognitive [16]. Environmental causes can include negative experiences in math classes or with particular math teachers [e.g., [17]]. Personal causes include low self-esteem, lack of confidence and the influence of previous negative experiences with mathematics [e.g., [18]]. Cognitive causes involve innate characteristics, being either low intelligence or simply poor cognitive abilities in mathematics [17]. Presently, we focus on the cognitive cause of math anxiety.

As noted above, there is evidence that math anxiety and math achievement are negatively correlated [e.g., [6,8,11]]. Very recently, Beilock and colleagues [7] have shown that girls' math achievement (but not boys') are influenced by their teachers math anxiety. If we wish to disentangle the relationship between the two, math achievement and anxiety, a logical first research step is to study math anxiety in individuals who suffer from low math abilities from very early in life (i.e., those with DD). To our knowledge, the relationship between math anxiety and developmental dyscalculia has received no formal attention from researchers, educators or clinicians. In the present paper, we aim to determine the precise relationship between math anxiety and DD.

## Developmental Dyscalculia

Developmental Dyscalculia (DD) describes a specific and severe deficit in the ability to process numerical information that cannot be ascribed to sensory difficulties, low IQ or inadequate education, and that results in a failure to develop fluent numerical computation skills [19,20]. Untreated, DD typically persists beyond the school-age years into late adolescence and adulthood [21,22]. Epidemiological studies indicate that DD is as common as reading disorders and affects 3.5% - 6.5% of the school-age population [20]. Moreover, DD runs in families and is heritable, which implicates genetic factors in its etiology, though to date none have been reported [23,24].

More recently, DD has been understood to reflect deficiency mainly (but not only) in brain regions of the parietal cortex along the intraparietal sulcus (IPS). IPS deficiencies can be found at the structural [25,26] and the functional levels (e.g., [DD in children [27-29], DD in adults [30,31]]) alike. The IPS is considered to be involved with an abstract, amodal representation of numbers [32-34] and is activated by numbers presented in different culturally learned symbolic notations such as Arabic numerals and spoken number words [35]. Accordingly, and despite indecisiveness in the existent development brain imaging studies on DD (e.g., for group differences on non-symbolic number processing in children with DD see [29] vs. no such difference in [28]), to date the IPS is the best validated core deficit of DD [21].

A range of terms are used to refer to problems in learning mathematical concepts and skills, including Math Difficulties, Math Disability, Mathematical Learning Disability, Mathematics Disorder, Specific Disorder of Arithmetic Skills, Math Anxiety, and DD. These terms are similar in that all implicate low numeracy skills. However, they are not synonymous [19,36]. Here we differentiate general Mathematics Disorder [MD: e.g., DSM-IV: [37]] from Developmental Dyscalculia in several important ways. In DD, the learning problem: 1) is specific to the domain of arithmetic (reading and spelling skills are within the normal range); 2) manifests partly as problems in learning and remembering simple arithmetic facts (such as single-digit sums or products; e.g., 3+4 = 7), rather than more general problems in computation; 3) is typically defined by very low scores on standardized tests of arithmetic achievement, e.g., below the 8th or even 5th percentile, which is equivalent to standard scores below 78 [38]; and 4) reflects a specific impairment in brain function that gives rise to unexpected problems in basic numerical processing, such as automatic or implicit processing of quantities or numbers [[28,30,39], e.g., [40-43]].

Further evidence of the distinction between DD and low numeracy is provided by our research, which shows the effects of a stimulant (methylphenidate) on arithmetic problem solving in children with ADHD+DD versus ADHD+ low numeracy. The drug had no influence on a basic understanding of numerical magnitude, which was impaired in the former but not the latter group [44]. It should be noted, as well, that the common symptom of poor memory for arithmetical facts in DD is not necessarily part of a wider impairment in either long-term or working memory [45,46].

The present study focuses on the narrower phenotype of DD rather than the broader diagnostic category of MD, since it is believed that the etiology of DD is distinct from that of other math difficulties [19,47]. As noted above, it was shown [48] that initial low mathematics achievement is related to later high mathematics anxiety (though primarily for boys; for girls this was true at critical transition points only). Although the relationship between math anxiety and DD has not yet been scientifically studied, Ma and Xu's findings [46] suggest that children with DD may find daily math lessons a source of huge anxiety, as they must exert tremendous effort to understand what is obvious to their classmates. This leads to our main hypothesis, namely, that DD is strongly associated with math anxiety. Our approach to this question combines the field of math anxiety [4] with that of mathematical cognition [49], which focuses on the underlying mental representations and processes used in arithmetic and mathematics performance.

## Affective priming and the current study

Typically, evidence shows significant deficiencies in performance as a function of math anxiety only when complicated arithmetic problems are tested [e.g., [50]]. These deficiencies have not been observed in simple operations such as single-digit addition or multiplication (e.g., 7 + 9, 6 X8), probably since the findings are usually based on paper-and-pencil behavioral tests that involve higher-level cognitive processes [e.g., [9]]. In the present study, we use a novel implicit low-level cognitive task [i.e., affective priming: for review see 51] to study math anxiety and its effects on math performance. We also use this task to study the differences between children with Developmental Dyscalculia and normally developing children.

It has been shown that affective traits can be activated automatically and influence emotional, cognitive or behavioral processes [e.g., [52]]. That is, affective processing starts immediately and even involuntarily upon seeing a salient affective word or picture. Psychologists use situations where implicit processing is possible in order to study automaticity. One such task is the priming task in which an early stimulus that is designed to be ignored influences the response to a subsequent relevant stimulus. In many cases, participants cannot ignore the irrelevant dimension (the prime), which facilitates or interferes with processing the relevant one (the target).

In line with these findings, affective priming studies have demonstrated that people respond to target stimuli more quickly after presentation of an affectively related prime stimulus than after one that is affectively unrelated, whether the target involves written words or not [for review see [51], e.g., naming target' written words: [53,54], naming or categorizing pictures: [55]].

One hypothesis is that affective priming works because affective polarized prime stimuli pre-activate the memory representation of affectively related targets [e.g., [56-58]]. For example, in naming tasks, words are automatically retrieved from verbal memory. This memory retrieval is influenced by the emotional prime through emotional coding associated with the target words [59]. In the present study, we ask whether single-digit arithmetic problems can be affectively primed in the same way. For example, it has been shown that with increasing practice or skill, children and adults automatically retrieve the solutions to simple addition [e.g., 3 + 4 = 7: [60,61]] and multiplication math problems [e.g., 3 × 4 = 12: [62,63]] from verbal memory as the strategy of choice and do not involve quantity processing. In contrast, single-digit subtraction and, sometimes, simple division appear to activate a distinct neural network that includes the inferior parietal lobule, left precuneus, and left superior parietal gyrus [64], suggesting that subtraction requires manipulation of mental quantities that is not automatic or implicit [65-68]. Recently, though, it has been shown [69] that in cases of very simple arithmetic operations presented with Arabic digits (as in the current study), most participants report using retrieval from memory as a strategy of choice not only for addition and multiplication but also for division and subtraction (about 82% use retrieval from memory for addition, 92% for multiplication, 80% for division and 73% for subtraction). It is not yet clear, however, to what extent simple addition or multiplication problems are affectively represented in verbal memory.

Accordingly, we developed for this study a novel task with four different types of primes (words with positive, neutral, and negative affect, as well as words related to mathematics) with single-digit arithmetic problems as targets. In our task, participants were simply required to decide if the target was true or false. This is similar to previous affective priming tasks in which participants were told to categorize the target as positive or negative [e.g., [59]]. It is hypothesized that in such cases, the affective priming effect is produced by processes that operate at a response selection level. Specifically, whenever a prime is affectively related to a target (e.g., both are positive), its valence will also match the valence of the response that is required (e.g., "positive," or in the current work, "true") [e.g., [59,70]]. Since it is not clear if emotional priming will influence math problems at the level of retrieval from verbal memory or at the level of response selection of computational procedures, we chose methodological parameters that have been proven to influence both levels [59] - namely, short stimulus onset asynchrony (SOA).

We use this novel arithmetic-affective priming task to study math anxiety, focusing on how the presentation of mathematical word-primes such as "multiplication" or "quantity" influences the ability to solve simple arithmetic problems, and how these math words compare in their effects to negative, positive, or neutral words. Importantly, we also study how performance of this task differs between children with DD and normally developing controls.

As noted above, in the current study we argue that math anxiety is related to poorer math performance, perhaps through the mechanism identified by Ashcraft and Kirk [see also [48,71]], who showed that the effect of math anxiety on performance is mediated by working memory. For this reason, we measured math anxiety solely through the arithmetic-affective priming paradigm (as opposed to using tools such as a math anxiety questionnaire). We used the Revised Manifest Anxiety Scale (RCMAS-2) [72] to measure general anxiety traits so as to distinguish those from math anxiety symptoms.

In this study, we used our novel affective-priming tool to address three hypotheses. We predicted that (1) a direct link would appear between emotions (primes) and arithmetic problem solving (targets); (2) compare to controls, DD will respond faster to targets that are preceded by negative affective primes that, in this group, will act as affectively related primes; and (3) math primes (e.g., words like "quantity") will have the same effect as the negative affective primes (i.e., acting as an affectively related primes) in the DD group but not in the controls, at least for some of the arithmetic problems (i.e., targets).

## Method

### Participants

A total of 36 children (aged 7 to13 years; 58% female) and their parents agreed to participate. Of these, 18 (50%) met our criteria for DD (see also Table 1). The DD and control groups were matched for age, IQ, reading ability and gender. Among the 36 participants, only 23 (12 with DD and 11 controls) completed all the trials (13 decided to quit before the task ended) in the arithmetic-affective priming task. These children comprise the final study sample. All participants in the final sample were in grades 4 or above.

**Table 1:** Characteristics of current sample (DD, controls)

Characteristic	DD	Controls	F (1,34)	*p*-value
Age (yrs)	10.6 (1.6)	9.6 (1.5)	3.87	ns (> 0.05)
Gender (% female)	61%	55%	Χ^2^	ns
K-BIT IQ (ss)	91.0 (18.9)	99.8 (19.5)	1.8	ns
*WJIII Math Computation (ss)	***84.2 (8.5)***	***95.6 (11.2)***	***12.3***	***.001***
*WJIII Math Fact Fluency (ss)	***71.6 (7.3)***	***92.2 (9.4)***	***53.9***	***.000***
*Dyscalculia Screener Dot Enumeration (stanine)^1^	***3.0 (1.5)***	***5.6 (1.7)***	***20.3*** ***(df: 1, 32)***	***.000***
*Dyscalculia Screener Number Comparison (stanine)	***3.8 (1.5)***	***5.3 (1.9)***	***6.8*** ***(df: 1, 32)***	***.01***
Dyscalculia Screener Addition (stanine)	3.3 (1.3)	3.8 (1.6)	< 1 (df: 1, 32)	ns
Dyscalculia Screener Simple Reaction Time (stanine)	2.8 (1.2)	3.9 (1.4)	6.7 (df: 1, 32)	.01
WJIII Word Attack (ss)	99.8 (11.4)	106.2 (10.5)	2.7	ns
WJIII Letter-Word Identification (ss)	95.4 (13.0)	103.8 (11.1)	3.9	ns (> .05)
WJIII Reading Fluency (ss)	94.4 (16.4)	105.9 (15.4)	4.5	ns
Conners Parent DSM-IV Inattention (T-score)	60.9 (10.4)	58.6 (13.1)	< 1	ns
Conners Parent DSM-IV Hyperactivity/Impulsivity (T-score)	52.2 (12.4)	57.2 (12.6)	1.4	ns
Conners Teacher DSM-IV Inattention (T-score)	61.1 (6.2)	55.5 (6.4)	5.7	.02
Conners Teacher DSM-IV Hyperactivity/Impulsivity (T-score)	57.9 (14.3)	52.4 (9.8)	1.3	ns
RCMAS Total Anx T-score	48.0 (11.4)	42 (10.4)	< 1	ns
Working Memory				
Cantab Spatial Span For (ss)	55 (0.8)	54 (1.0)	< 1	ns
Cantab Spatial Span Back (ss)	60 (0.7)	68 (.7)	2.1	> .1
Letter Span Forward (raw)	3.6 (1.1)	3.4 (.9)	< 1	ns
Letter Span Backward (raw)	1.6 (0.8)	1.6 (.8)	< 1	ns

* Four tests used to define Dyscalculia, plus IQ and reading in normal range

^1^Stanine scores: stanine score of 1 = standardized score [SS] < 74; 2 = SS 74-81; 3 = SS 82-88; 4 = SS 89-96; 5 = SS 97-103; 6 = SS 104-111, and so on.

Both groups of children were recruited from two private schools in Canada, which accepted students with severe mathematical learning difficulties, as well as typically achieving students. Teachers were asked to nominate students who they believed were of average general ability but had serious difficulties with numeracy. For each child classified as having DD (according to assessment criteria described below), we recruited a typically achieving student of same age and gender from the same class.

#### General inclusion/exclusion criteria

All children spoke English as their primary language, attended school full time, had no uncorrected sensory or physical impairments (which would preclude participation in the computerized or paper-and-pencil testing), and no current or previous history of psychosis or other mental health disorders (e.g., ADHD, Anxiety, or Depression) that might influence cognitive performance.

*Classification of DD *(see Appendix 1 for description of classification measures)

To be classified as having DD, children had to meet the following three criteria:

(1) at least average general ability, as indexed by standardized scores of at least 85, or 16th percentile, on the Kaufman Brief Intelligence Test (K-BIT2) [73];

(2) a learning problem specific to the domain of arithmetic, as shown by scores of normal range (≥ 85, or 16th percentile) on three standardized reading tests - *Word Attack, Letter-Word Identification*, and *Reading Fluency *- from the Woodcock-Johnson Tests of Achievement, 3^rd ^Edition (WJ-III) [74];

(3) impaired numeracy skills as indexed by standardized scores ≤ 81 (below 10^th ^percentile) or a stanine score < 3 on at least one of two subtests of the WJ-III (*Math Computation *and *Math Fluency) *and on at least one of the two subtests from the Dyscalculia Screener [75]: Dot Enumeration and Number Comparison, which are considered core subtests for identifying DD [47,75];

(4) no signs for generalized anxiety traits according to the Revised Manifest Anxiety Scale (RCMAS-2).

(5) no signs of working memory impairments according to Cambridge Neuropsychological Testing Automated Battery (CANTAB) and letter span.

To be classified as typically achieving controls, children had to meet the following three criteria:

(1) no parent or teacher concerns regarding academic achievement, attention, or behavior (as indexed by the Academic Screening Rating Scale [76];and SWAN rating scale [77];

(2) at least average general ability, as indexed by standardized scores of at least 85 or 16th percentile, on the K-BIT2 [73];

(3) At least average reading and numeracy skills as indexed by standardized scores of at least 85 (above 16^th ^percentile) or a stanine score of at least 4 on the two reading tests together with four math tests described above (i.e., score of 85 and above was required on all 6 tests);

(4) no signs for generalized anxiety traits according to the Revised Manifest Anxiety Scale (RCMAS-2).

(5) no signs of working memory impairments according to Cambridge Neuropsychological Testing Automated Battery (CANTAB) and letter span.

### Arithmetic-affective priming task

#### Stimuli

Each trial consisted of a prime (one of the four types of affective words) and target (simple arithmetic equation) that appeared sequentially. Both prime and target appeared horizontally at the center of a computer screen in black characters against a white background. Each character was printed in boldface in Ariel font, size 18.

#### Primes

A list of 40 words (10 negative, 10 positive, 10 neutral, and 10 mathematics words) comprised the primes (see Appendix for details of the primes). Valences for the emotional and neutral words were taken from Vasa and colleagues' data [78]. Word frequencies shown in the Additional file 1 (Appendix 2) are taken from [79] and, for the emotional and neutral words, also from [80]. Note that there are no norms for emotional values of mathematical words.

#### Targets

Equations were presented in the form "a * b = c ", where a and b represented single digits from 1 to 9, * represented an arithmetic operation (×, +, -, or ÷), and c represented the solution. We employed for a and b all possible pairs of digits from 1 to 9 such that (1) regardless of the arithmetic operation used, the solution to the equation is a positive integer; and (2) the four arithmetic operations produce different solutions. For example, 7 * 3 and 5 * 4 were excluded because in these cases, division results in a solution that is not an integer. Likewise, we excluded equations such as 3 * 1, where multiplication and division produce the same result, and 4 * 2, where this is true for division and subtraction. Five pairs of digits meet both criteria (9 * 3, 8 * 4, 8 * 2, 6 * 3, and 6 * 2), and so these pairs were included in the experimental blocks. For all stimuli, the numerically larger digit was presented on the left side.

We created two types of solution conditions as follows: (1) the true condition comprised 20 equations with true results according to the criteria described above (e.g., 8 × 4 = 32); (2) for each of the 20 true equations we created three false equations by borrowing the solution to another equation (as long as this solution was not the same as a or b, i.e., a digit belonging to the arithmetic fact itself). For example, for the stimulus 8 × 4, we created false equations with the solutions 27, 10 and 3, which are true results for 3 × 9, 8 + 2 and 6 ÷2, respectively. (See Additional file 1: Appendix 3 for the target arithmetic problems).

Each participant underwent 160 trials using the 40 primes four times each, twice with a true equation as the target and twice with a false equation. For the true condition, each prime appeared with two different true equations that were pseudo-randomly selected such that any given true equation appeared only once for each group of 10 primes. This produced a total of 80 true equations.

For the false condition, each prime appeared with two different false equations, which again were pseudo-randomly selected such that any given false equation appeared once in each group of 10 primes. This produced a total of 80 false equations. The distribution of false equations was designed such that each participant saw only one of the three different false equations that had been created for every true one, but that all the false equations were used. In other words, the full set of 60 false equations was seen by each set of three participants together.

The following two variables were included in the analysis: group (DD vs. control), prime (negative, positive, neutral, and math), and target's arithmetic operation (+, -, ×, ÷). Thus, we had a 2 × 4 × 4 factorial design. Group was the only between-participants variable and primes and targets were manipulated within block.

Before the experiment began, participants completed a practice phase with eight primes (see Additional file 1: Appendix 4) and eight equations, four true and four false (see Additional file 1: Appendix 3). The primes and equations were different from those used in the experiment itself.

### Procedure

Stimuli were presented on a computer screen at a distance of approximately 60 cm from participants. Participants were told that they were about to participate in a simple arithmetic experiment and that a word and simple arithmetic problem would be sequentially presented on the computer screen. They were instructed to decide if the arithmetic problem was correct or not as quickly as possible while ignoring the word. Reponses were made by pressing one of two possible keys.

Each trial opened with a 500 millisecond (ms) presentation of a fixation cross in the center of the computer screen. Five hundred milliseconds after offset of the fixation cross, the prime words were presented for 250 ms. The target arithmetic equation followed an offset of the prime words, resulting in a stimulus onset asynchrony (SOA) of 250 ms. The target equations were displayed until the participant responded "correct" or "not correct" by pressing one of two keys on the keyboard (the letters "p" or "q"), or until 3,000 ms had elapsed. The correct solution was represented by the letter "p" for 14 participants and the letter "q" for the other nine. The next trial was initiated 2,000 ms after the participant's response. Reaction time (RT) in milliseconds was measured by the computer from the stimulus onset to the participant's response.

## Results

Incorrect responses (3.3%) were discarded from the analysis. Also, to reduce the impact of outliers, response latencies that deviated more than 2.5 standard deviations from the participant's conditional mean latency (0.7%) were discarded, as well. An accuracy analysis demonstrated that the number of errors made by the DD group did not differ significantly from those made by the control group, *F*(1,10) = 4.566, *p *= 0.058. Also, the correlation between error rates and RT was positive *r = *0.4399, *p *< 0.05 thereby, excluding any speed-accuracy tradeoff.

The main effects of group (DD: M = 977.8 ms, S.D. = 29.8 ms; Control: M = 844 ms, S.D. = 20.1 ms); *F*(1,21) = 2541, *p *< 0.001, type of prime (positive primes: M = 920.7 ms, S.D. = 89.9 ms; neutral: M = 912.3 ms, S.D. = 69.3 ms; negative: M = 912 ms, S.D. = 61.41 ms; math: M = 909 ms, S.D. = 63.24 ms; *F*(3,63) = 31, *p *< 0.001) and arithmetic operation (i.e., target; addition: M = 896.6 ms, S.D. = 66.64 ms; multiplication: M = 899.9 ms, S.D. = 67.96 ms; subtraction: M = 923.2 ms, S.D. = 70.62 ms; division: M = 935.5 ms, S.D. = 74.9 ms; F(3,63) = 43.2, p < 0.001), reached significance.

Of primary relevance to the aims of the current study was the significant interaction between group, type of prime and arithmetic operation, *F*(9,189) = 10.4, p < 0.001 (see Table 2). Planned comparisons, based on our initial hypothesis, confirmed that the simple interaction between type of prime × arithmetic operation was significant in the control group, *F*(9,90) = 4.06, *p *< 0.01, as well as in the DD group, *F*(9,99) = 9.43, *p *< 0.001. However, as predicted, further planned comparisons showed that differential priming (relatedness) effect was obtained in the priming data. Specifically, analysis revealed an affective priming effect (i.e., positive affective primes vs. negative affective primes) in the control group. That is, a significantly shorter response latencies for positive as compared to negative affective primes was found in the control group (i.e., positive affective primes tend to act as affectively related to simple arithmetic problems) when targets were addition problems, *F*(1,10) = 47.1, *p *< 0.001; multiplication, *F*(1,10) = 54.23; *p *< 0.001, subtraction, *F*(1,10) = 799.9, *p *< 0.001; and division problems, *F*(1,10) = 905, *p *< 0.001(see Table 2 for further details).

**Table 2:** Means for reaction times (RT) as a function of the difference between prime valence and target

		Control		DD	
**Target**	**Prime valence**	**Mean RT in ms**	**F(1,10) =**	**Mean RT in ms**	***F*(1,11) =**

**Addition**	**Positive - Negative**	-24	47.1, p < 0.001	38	259, *p *< 0.001
	**Positive - Neutral**	-13	162, p < 0.001	26	48.3, p < 0.01
	**Positive - Math**	-14	30. 9, *p *< 0.001	34	52, *p *< 0.001
	**Negative - Neutral**	11	8.82, p < 0.05	-12	21.3, *p *< 0.01
	**Negative - Math**	10	8.1, *p *< 0.05	-4	n.s.
	**Math - Neutral**	1	n.s.	-8	n.s.

**Multiplication**	**Positive - Negative**	-26	54.2, *p *< 0.001	38	902, *p *< 0.001
	**Positive - Neutral**	-13	122.1, *p *< 0.001	13	328.3, *p *< 0.001
	**Positive - Math**	-16	146.2, *p *< 0.001	36	253.9, *p *< 0.001
	**Negative - Neutral**	13	10.86, *p *< 0.05	-15	192.5, *p *< 0.001
	**Negative - Math**	10	7.01, *p *< 0.05	-2	n.s.
	**Math - Neutral**	2	n.s.	-13	18.9, *p *< 0.01

**Subtraction**	**Positive - Negative**	-23	799.9, *p *< 0.001	55	119.7, *p *< 0.001
	**Positive - Neutral**	-13	94.2, *p *< 0.001	40	227, *p *< 0.001
	**Positive - Math**	-18	100.3, *p *< 0.001	40	226, *p *< 0.001
	**Negative - Neutral**	10	23.5, *p *< 0.01	-15	80.14, *p *< 0.001
	**Negative - Math**	5	5.35, *p *< 0.05	-15	16.9, *p *< 0.01
	**Math - Neutral**	5	23.6, *p *< 0.01	0	n.s.

**Division**	**Positive - Negative**	-23	905, *p *< 0.001	19	625, *p *< 0.001
	**Positive - Neutral**	-15	103, *p *< 0.001	25	65.3, *p *< 0.001
	**Positive - Math**	-27	237 *p *< 0.001	42	98.9, *p *< 0.001
	**Negative - Neutral**	8	28.6, *p *< 0.001	6	n.s.
	**Negative - Math**	-4	n.s.	23	28.5, *p *< 0.01
	**Math - Neutral**	12	23.7, *p *< 0.001	-17	10.6, *p *< 0.05

An affective priming effect was found in the DD group, as well, but with a reversed pattern. That is, significantly shorter response latencies were observed in the DD group for negative as compared to positive targets (i.e., *negative *affective primes act as affectively related to simple arithmetic problems) when targets were addition problems, *F*(1,11) = 259.2, *p *< 0.001; multiplication, *F*(1,11) = 902; *p *< 0.001, subtraction, *F*(1,11) = 1197, *p *< 0.001; and division problems, F(1,11) = 625, p < 0.001. Accordingly, the affective priming effect in the DD group (i.e., related\negative primes vs. unrelated\positive primes) is different from that found in the control group (i.e., related\positive primes vs. unrelated\negative primes)

**Figure 1:**
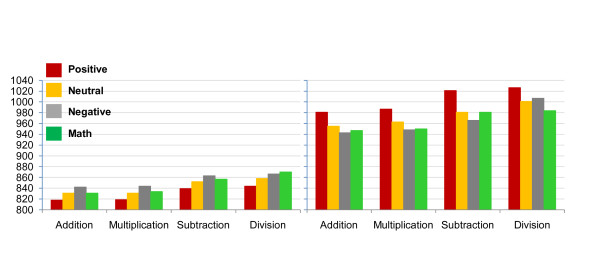
**Response times to addition, multiplication, subtraction, and division problems in the control (left panel) and dyscalculia (DD; right panel) groups, as a function of the affective primes**.

We further contrasted the results for the four different primes in relation to each arithmetic operation for each group (see Table 2 and Figure 1). In general, we found that in **the DD group **there was no significant difference between negative and math primes for addition and multiplication. Furthermore, in the case of division, math prime words facilitated processing even more than the negative prime words (target's RTs were shorter after presentation of math prime words compared to negative prime words) and hence, acted as affectively related to targets (even more than negative emotional primes). Supporting this last claim is the finding that in all 4 arithmetic procedures, math prime words significantly facilitated processing compared to positive prime words. An opposite pattern was found in **the control group**: In 3 arithmetic procedures (addition, multiplication and subtraction) responding to targets were faster after the presentation of math prime words compared to the presentation of negative prime words but still significantly slower compared to when being presented after positive prime words. In the case of division (in the control group), math prime words inhibited processing (i.e., response latencies to targets were shorter after presentation of math prime words as in the case of negative prime words) and hence, acted as affectively unrelated to targets similar to negative emotional primes. Hence, in the control group, math prime words acted as an affectively unrelated to targets. In addition, in the control group we found no significant difference between neutral and math word primes for addition and multiplication.

## Discussion

Data from behavioral and cognitive studies with typically developing participants have long implicated the role of low self esteem and anxiety in arithmetic and math [[48], e.g., [71]]. It appears that poor math achievement is strongly related to math anxiety [6-8,11,48,50]. However, there has thus far been no direct evidence linking math anxiety with deficiencies in basic numerical abilities (e.g., retrieval of arithmetic facts). In the present study, we evaluated the relationship between math abilities, math anxiety and simple arithmetic problems by using novel arithmetic-affective priming during simple arithmetic problem solving. Analyses of the data revealed that participants with DD responded faster to targets that were preceded by both negative primes and math-related primes. A reversed pattern was present in the control group. We found this effect for each of the arithmetic procedures (multiplication, addition, subtraction and division), thereby uncovering a direct link between emotion and simple arithmetic problem solving, and more specifically, between negative emotions and deficits in simple arithmetic problem solving in cases of DD. Moreover, we also found simple arithmetic problem solving to be modulated by words that are related to math (e.g., "quantity"). In the DD group math words and negative affective primes had a significantly similar effect on targets. Specifically, in both addition and multiplication problems as targets, math prime words had precisely the same facilitative influence as negative affective words. Moreover, in all four procedures, math prime words facilitated target processing in a similar fashion as the influence of negative primes (in divisions, facilitation effect was even stronger). This suggests that these math words are affectively related to targets (which are loaded by negative affect). However, in the control group, math words inhibited processing. Specifically, in both addition and multiplication problems as targets, math prime words had the same influence as neutral affective words; and in all four procedures math prime words inhibit processing similar (or even more) to the influence exerted by the negative affective primes. This suggests that, in the control group, these math words are affectively unrelated to targets (which may be loaded by neutral affect).

### Math anxiety and math achievement

The main contribution of the current work is that it provides significant support for the proposed relationship between math anxiety and math achievement [e.g., [17]]. We found a strong relationship between DD (i.e., severe math difficulties) and fear, especially when children in this group were required to decide quickly whether problems in all four procedures were correct or not.

The following is only a suggested hypothesis, since the current analysis cannot point directly to a causal connection between math anxiety and math achievement. Rubinsten and Henik [19] have recently suggested frameworks to link the behavioral-cognitive deficits associated with DD to their potential neural foundations. In general research suggests that DD has a clear biological source and reflects deficiency mainly in brain regions of the parietal cortex along the IPS. Hence, and if DD is a heritable, genetically-based condition [e.g., see recent genetic study by [81]], then youngsters with DD likely suffer from low numerical abilities from a very early age. This strongly suggests that low math achievement due to DD will lead to math anxiety. To some extent, our findings support Ma and Xu's [48] data, suggesting that math anxiety springs from the unpleasant memory of poor mathematics performance in the past. Accordingly, it seems reasonable to assume that most of the evaluative reactions towards math are learned rather than innate.

According to our data, there is clear evidence that, for DD, math words had an anxious influence mainly when it comes to addition and multiplication arithmetic problems. What may be some of the reasons for this phenomenon? Normally developing children enter school with informal knowledge about numbers and arithmetic; knowledge that is based on their daily experiences of counting and calculation. Once entering school, however, much educational training is focused on basic multiplication and addition arithmetic facts [82]. Consider, however, a child with DD who is innately deficient in his/her ability to process numbers, to count and to calculate. This child, from a very young age, has to answer addition and multiplication questions for which there is almost always only one correct answer. This situation, combined with the culture of solving these problems quickly, can lead students with DD towards a negative attitude style and ultimately learned helplessness to arithmetic in general (i.e., the affectively related influence that negative affective words had on solving simple arithmetic problems). Also, this situation can lead to a specific and accentuated fear and avoidance when it comes to retrieval of addition and multiplication problems from memory (i.e., the affectively related influence that math words had on solving mainly multiplication and addition problems). Recently, Ischebeck and colleagues [83] found that untrained complex multiplication problems produce activation in several frontal and parietal brain areas. With training, activation shifts to the left angular gyrus, known to be involved in retrieval of arithmetic facts. Importantly and with relevance to the current study, only division problems (that were not trained) that are related to the trained multiplication problems showed also activation in the left angular gyrus. This may suggest that there is a transfer of knowledge and procedure form multiplication to division. Taken together data from Ischebeck and our study, suggest that anxiety and fear are similarly associated with division problems just as they are associated with multiplication problems.

### Affective priming: Methodological issues

It should be noted that recent research on affective priming has primarily focused on the contributions of valence relatedness to priming, i.e., where positive prime and negative target and vice versa are considered as unrelated trials and positive prime and target or negative prime or target are considered as related trials. [for review see [51]]. However, in the current work, the affective value of the target (a simple arithmetic problem) was initially unknown and actually was studied. Hence, relatedness could not be established. Accordingly, and in contrast with previous affective priming studies, we were interested not in affective relatedness but rather in the specific influence of positive versus negative affect on arithmetic performance.

We found that in the DD group responding to targets was faster after a negative affective prime in comparison to a positive affective prime. Similar results were found for math-related prime words. Considering that it is typically found that people respond to target stimuli more quickly after presentation of an affectively related prime than after one that is affectively unrelated, it may be suggested that for the DD group, arithmetic facts are associated with negative emotions as well as math related words [for review see [51], e.g., naming target' written words: 53, 54, naming or categorizing pictures: 55]. Our results support recent theoretical models which suggest, for example, that positive affect promotes associations between strong and weak concepts, and that negative affect impairs such associations [84]. Therefore, the current arithmetic-affective priming might be a useful tool in establishing the affective value of various arithmetic procedures in different types of math-achieving participants.

The current findings also suggest that math words indeed endorse math anxiety in DD. Hence, since math word primes, in particular, were designed to elicit math anxiety, the data strongly suggests that indeed these math words did evoke math anxiety.

It should be noted that Ashcraft and Krause [13] indicated that working memory resources in math-anxious individuals are drained only when math anxiety is aroused. Thus, it is essential to first highlight the finding that the DD and control groups in the current study did not differ in baseline working memory capacity (see Table 1 for working memory characteristics of the samples). Accordingly, group differences in effects of affective priming were found in the absence of any group differences in working memory. Also, it should be noted that both DD and controls have been recruited from the same schools. Hence, the differences in the affective priming effect are not the results of socioeconomic background since both groups of participants came from the same socioeconomic background (middle-class). Moreover, by using the arithmetic-affective priming task, we were able to show that math anxiety is specific to the math context, and so is distinct from and occurs in the absence of generalized anxiety. Last, general negative attitude (towards everything), could not lead to a specific negative influence of only math words in children with DD. In case of general negative attitude one should expect that positive words would have a similar negative influence in DD. This was not found. All of this indicates that affective priming effects could be attributed to math anxiety per se. As was previously shown [e.g., [6]], math anxiety manifests itself as an unpleasant emotional response to math. This is what we show here - simple arithmetic and math words are associated with unpleasant emotions. Accordingly, it may be suggested that our arithmetic-affective priming may be used as an indirect measure of math anxiety.

### Significance, limitation and suggestions

Since it was first described by Richardson and Suinn and colleges in 1972, math anxiety has rarely been the subject of inquiry. This is true despite the fact that an understanding of the effects of math anxiety is fundamental to our understanding of the human cognitive apparatus in numerical abilities. Moreover, in the field of math anxiety, findings are usually based on paper-and-pencil behavioral tests that involve higher-level cognitive processes and, therefore, cannot provide a detailed description of the phenomena. By using the novel numerical affective priming, we were able to provide definitive evidence that, for DD children, arithmetic is related with fear.

Regarding the current results, it is clear that math achievement tests are not genuine measures of math achievement. Specifically, it is possible that children with high math anxiety achieve low scores on math achievement tests because their anxiety interferes while they do the test. It should be emphasized, though, that the results of the Revised Manifest Anxiety Scale (RCMAS-2) [72] were similar for the DD and control groups in our study, suggesting no general anxiety in either group.

Hembree [6] showed that cognitive-behavioral interventions for math anxiety had a positive influence on math achievement test scores. These findings are quite significant in terms of the relationship between math anxiety and math achievement, and specifically in relation to DD. For people with DD, childhood difficulties with numerical processes and poor math achievement intensify math anxiety, which further impedes math achievement. As educators come to appreciate the key role played by math anxiety, interventions that reduce it may become a key part of the math educational system. It might be that one of the most effective ways to reduce math anxiety is to improve math achievement from an early age through interventions focused on children with DD thus turning the cycle of failure-fear-failure to one of success-confidence-success. This is especially true if the assumption that DD is an innate condition is correct. Such programs would be an important way of helping students cope with the frustrations inherent in the learning of mathematics, and thereby improve math achievement.

## Competing interests

The authors declare that they have no competing interests.

## Authors' contributions

OR contributed to conception and design of the study, took care of the data acquisition, performed the data analyses and interpretation and drafted the manuscript. RT contributed to the design of the study, took care of the data acquisition, participated in data interpretation, helped drafting the manuscript and critically revised the results. All authors read and approved the final manuscript.

## Appendix

### Appendix 1

#### Classification Measures

i) Kaufman Brief Intelligence Test, Second Edition (KBIT-2) [73]. The KBIT-2 is a brief and validated individually administered measure of verbal and non-verbal intelligence that yields an estimated general IQ score. The test consists of two main subtests. The *Verbal Knowledge *subtest measures receptive vocabulary and general information about the world (by asking the child to point to a picture that best illustrates the specified word). The *Matrices *subtest measures the child's ability to solve new problems, perceive relationships and complete visual analogies without testing vocabulary or language skill (the child is asked to point to the picture that will complete a pattern).

ii) Woodcock-Johnson Tests of Achievement (3^rd ^Ed: WJIII ACH). We used five subtests from this well-validated achievement test. Two (*Math Computation *and *Math Fluency*) are widely used in Canada to help diagnose DD, and three (*Letter-Word Identification, Word Attack*, and *Reading Fluency*) are used to rule out reading disabilities.

iii) Dyscalculia Screener [75]. We used two item-timed subtests from this standardized software: a dot enumeration task and a number comparison task, which constitute a 'capacity subscale', used to help classify DD. The dot enumeration task, which assesses the capacity to represent exact numerosities, requires the child to count the number of dots (ranging from 1 to 9) arrayed randomly in a box on half of the screen and determine whether the amount matches the Arabic numeral (1 to 9) presented on the other half of the screen. Responses (yes, no) to each of the 68 displays are indicated by button press. The number comparison task assesses the capacity to order numerosities by magnitude and understand the numerals. The child is presented with 42 sets of 2 black Arabic digits (1 to 9), which vary in physical size, and is required to indicate by button press which is the numerically larger of the two numerals. Scores are reported in stanines: a score in the lowest stanines (stanine 1 or 2, corresponding to standard scores < 81) on at least one of these two subtests is indicative of DD [47,75].

iv) Conners Rating Scale-Revised (CRS) [85]. Parents and teachers were asked to complete the relevant version of these scales to screen for ADHD. Children with T-scores ≥ 70 from one informant plus a T-score ≥ 60 from the other informant were classified as positive for ADHD and excluded.

v) Revised Manifest Anxiety Scale (RCMAS-2) [72]. We used the short form of this self-report scale for children that screens for the level and nature of trait anxiety, using a simple yes-or-no response format.

vi) Cambridge Neuropsychological Testing Automatized Battery (CANTAB) Spatial Span (Luciana, 2003): This computerized task assesses visuo spatial working memory and is a two-dimensional version of the Corsi Block Task. Nine white squares are presented on the screen, some of which momentarily change in color in a variable sequence. The participant must touch each of the boxes in the same (forward) or opposite (backward) order as they were colored by the computer. The number of boxes that change color (i.e., difficulty level) is increased from two to a maximum of nine. If the participant fails to replicate the correct sequence, the next trial remains at the same difficulty level. After three consecutive incorrect replications, the test is discontinued.

vii) Letter Span (adapted from the WISC-III-PI: Kaplan et al., 1999) to assess phonological/auditory working memory: This test is the same in format and administration procedure as the digit span task but uses alphanumeric letters rather than digits. Like the digit span task, each sequence length has two trials, with each trial in a series consisting of the same number of letters; however, the first of the two trials for each series consists of non-rhyming letters (e.g., X-R- S) whereas the second trial consists of rhyming letters (e.g., E - P - G).

## Supplementary Material

Additional file 1 **Appendices 2-4**. Appendix 2. Description of primes: stimuli words and relevant norms in the experimental block. Appendix 3. Description of targets - The simple arithmetic problems used on both the practice phase and experiment. Appendix 4. Description of primes: Stimuli words and norms in the practice phase. Click here for file
