# Supplementary material for: Mathematics anxiety in children with developmental dyscalculia
Source: Behav Brain Funct. 2010 Jul 15;6:46. doi: 10.1186/1744-9081-6-46 (PMC2913999; doi:10.1186/1744-9081-6-46)
Supplement: Additional file 1 — Appendices 2-4. Appendix 2. Description of primes: stimuli words and relevant norms in the experimental block. Appendix 3. Description of targets - The simple arithmetic problems used on both the practice phase and experiment. Appendix 4. Description of primes: Stimuli words and norms in the practice phase. [file 1744-9081-6-46-S1.DOC]

Appendix 2.

| **Emotionally negative words** | | | | | | | | **Emotionally neutral words** | | | | | | | |
| --- | --- | --- | --- | --- | --- | --- | --- | --- | --- | --- | --- | --- | --- | --- | --- |
|  | Valence rating | | | Word characteristics | | | |  | Valence rating | | | Word characteristics | | | |
| Word | Total -average (SD) ^b^ | Female ^b^ | Male ^b^ | Length | Frequency (Zeno et al., 2006) | Frequency (Nelson & McEvoy, 1998) | Concreteness  (Nelson & McEvoy, 1998) | Word | Total average (SD) ^c^ | Female ^c^ | Male ^c^ | Length | Frequency (Zeno et al., 2006) | Frequency (Nelson & McEvoy, 1998) | Concreteness  (Nelson & McEvoy, 1998) |
| **War** | 1.44 (0.41) | 1.29 | 1.60 | 3 | 163.25 | 464 | 4.73 | **Wall** | 3.10 (0.60) | 3.07 | 3.13 | 4 | 171.5 | 160 | 5.85 |
| **Fail** | 1.63 (0.76) | 1.49 | 1.76 | 4 | 12.5 | 37 | 3.23 | **Tray** | 3.13 (0.58) | 3.06 | 3.21 | 4 | 15 | 18 | 5.86 |
| **Hurt** | 1.74 (0.77) | 1.65 | 1.84 | 4 | 100 | 37 | 3.66 | **Wood** | 3.13 (0.55) | 3.11 | 3.15 | 4 | 171.5 | 55 | 5.81 |
| **Afraid** | 1.84 (0.64) | 1.77 | 1.89 | 6 | 127 | 57 | 3.32 | **Paper** | 3.18 (0.6) | 3.18 | 3.18 | 5 | 224 | 16 | 0 |
| **Stranger** | 1.91 (0.82) | 1.72 | 2.11 | 8 | 33.5 | 40 | 5.55 | **Jacket** | 3.4 (0.75) | 3.43 | 3.38 | 6 | 27.25 | 33 | 6.31 |
| **Stupid** | 2.05 (0.86) | 1.93 | 2.18 | 6 | 19.25 | 24 | 3.39 | **Pencil** | 3.18 (0.59) | 3.19 | 3.18 | 6 | 29 | 34 | 7 |
| **Lonely** | 2.06 (0.77) | 2.06 | 2.07 | 6 | 35.25 | 25 | 2.53 | **Plate** | 3.16 (0.51) | 3.12 | 3.19 | 5 | 35.25 | 22 | 5.74 |
| **Gun** | 1.45 (0.8) | 1.18 | 1.73 | 3 | 44.25 | 118 | 6.08 | **Block** | 3.05 (0.64) | 2.99 | 3.11 | 5 | 49.5 | 66 | 5.93 |
| **Mistake** | 2.41 (0.79) | 2.34 | 2.48 | 7 | 28.5 | 34 | 2.7 | **Article** | 3.26 (0.68) | 3.28 | 3.25 | 7 | 29.5 | 68 | 5.82 |
| **Dumb** | 2.03 (0.87 | 1.91 | 2.16 | 4 | 11.5 | 13 | 3.36 | **Statue** | 3.3 (0.68) | 3.29 | 3.31 | 6 | 16 | 15 | 6 |

Valence scale: 5=very happy; 4=somewhat happy; 3= neutral, 2=somewhat sad/scared, 1= very sad/scared

Ratings (within the same type of rating) with different superscripts differ significantly (p<.001).

Appendix 2. Continued.

| **Emotionally positive words** | | | | | | | | **Mathematics words** | | | | | | | |
| --- | --- | --- | --- | --- | --- | --- | --- | --- | --- | --- | --- | --- | --- | --- | --- |
|  | Valence rating | | | Word characteristics | | | |  | Valence rating | | | Word characteristics | | | |
| Word | Total average (SD) ^a^ | Female ^a^ | Male ^a^ | Length | Frequency (Zeno et al., 2006) | Frequency (Nelson & McEvoy, 1998) | Concreteness  (Nelson & McEvoy, 1998) | Word | Total | Female | Male | Length | Frequency (Zeno et al., 2006) | Frequency (Nelson & McEvoy, 1998) | Concreteness  (Nelson & McEvoy, 1998) |
| **Party** | 4.78 (0.51) | 4.81 | 4.75 | 5 | 91.25 | 215 | 5.5 | **Quantity** | NA | NA | NA | 8 | NA | 33 | 3.32 |
| **Kiss** | 4.01 (1.15) | 4.3 | 3.48 | 4 | 9.5 | 17 | 5.78 | **Math** | NA | NA | NA | 4 | NA | 4 | 3.78 |
| **Safe** | 4.66 (0.64) | 4.76 | 4.55 | 4 | 89 | 58 | 3.72 | **Divide** | NA | NA | NA | 6 | NA | 14 | 3.36 |
| **Pretty** | 4.16 (0.98) | 4.65 | 3.64 | 6 | 119.5 | 107 | 2.9 | **Subtract** | NA | NA | NA | 8 | NA | 2 | 2.86 |
| **Peace** | 4.66 (0.61) | 4.75 | 4.57 | 5 | 49.5 | 198 | 2.98 | **Add** | NA | NA | NA | 3 | NA | 88 | 2.95 |
| **Smile** | 4.66 (0.58) | 4.81 | 4.51 | 5 | 76.5 | 68 | 3.9 | **Multiply** | NA | NA | NA | 8 | NA | 10 | 3.55 |
| **Holiday** | 4.78 (0.51) | 4.82 | 4.73 | 7 | 14.75 | 17 | 3.6 | **Minus** | NA | NA | NA | 5 | NA | 8 | 2.75 |
| **Smart** | 4.78 (0.47) | 4.88 | 4.67 | 5 | 26.5 | 21 | 3 | **Number** | NA | NA | NA | 6 | NA | 472 | 3.76 |
| **Treasure** | 4.71 (0.57) | 4.67 | 4.74 | 8 | 29.5 | 4 | 5.05 | **Count** | NA | NA | NA | 5 | NA | 49 | 3.42 |
| **Hug** | 4.38 (0.71) | 4.62 | 4.13 | 3 | 5.25 | 3 | 5.34 | **Plus** | NA | NA | NA | 4 | NA | 52 | 2.18 |

Valence scale: 5=very happy; 4=somewhat happy; 3= neutral, 2=somewhat sad/scared, 1= very sad/scared, NA + Not applicable

Ratings (within the same type of rating) with different superscripts differ significantly (p<.001).

Appendix 3.

| **Targets in Experiment:**  **Correct equations** | **Targets in Experiment:**  **False equations** | **Targets in Practice:**  **Correct equations** | **Targets in Practice:**  **False equations** |
| --- | --- | --- | --- |
| 6-2=4 | 6-2=27 | 1+2=3 | 2+3=7 |
| 6-3=3 | 6-2=9 | 5-1=4 | 3+1=5 |
| 6÷2=3 | 6-2=10 | 4÷2=2 | 2+7=4 |
| 6÷3=2 | 6-3=27 | 2X3=6 | 3+5=12 |
| 6+2=8 | 6-3=32 |  |  |
| 6+3=9 | 6-3=4 |  |  |
| 6X2=12 | 6÷2=10 |  |  |
| 6x3=18 | 6÷2=18 |  |  |
| 8-2=6 | 6÷2=9 |  |  |
| 8-4=4 | 6÷3=10 |  |  |
| 8÷2=4 | 6÷3=16 |  |  |
| 8÷4=2 | 6÷3=8 |  |  |
| 8+2=10 | 6+2=10 |  |  |
| 8+4=12 | 6+2=16 |  |  |
| 8x2=16 | 6+2=2 |  |  |
| 8x4=32 | 6+3=4 |  |  |
| 9-3=6 | 6+3=8 |  |  |
| 9÷3=3 | 6+3=2 |  |  |
| 9+3=12 | 6x2=2 |  |  |
| 9x3=27 | 6x2=9 |  |  |
|  | 6X2=10 |  |  |
|  | 6x3=10 |  |  |
|  | 6x3=4 |  |  |
|  | 6x3=8 |  |  |
|  | 8-2=27 |  |  |
|  | 8-2=3 |  |  |
|  | 8-2=32 |  |  |
|  | 8-4=18 |  |  |
|  | 8-4=3 |  |  |
|  | 8÷2=18 |  |  |
|  | 8÷2=2 |  |  |
|  | 8÷2=9 |  |  |
|  | 8÷4=16 |  |  |
|  | 8÷4=18 |  |  |
|  | 8÷4=6 |  |  |
|  | 8+2=12 |  |  |
|  | 8+2=3 |  |  |
|  | 8+2=16 |  |  |
|  | 8+4=27 |  |  |
|  | 8+4=3 |  |  |
|  | 8+4=6 |  |  |
|  | 8x2=27 |  |  |
|  | 8x2=3 |  |  |
|  | 8x2=9 |  |  |
|  | 8x4=16 |  |  |
|  | 8x4=3 |  |  |
|  | 8x4=6 |  |  |
|  | 9-3=2 |  |  |
|  | 9-3=32 |  |  |
|  | 9-3=16 |  |  |
|  | 9÷3=10 |  |  |
|  | 9÷3=18 |  |  |
|  | 9÷3=4 |  |  |
|  | 9+3=16 |  |  |
|  | 9+3=2 |  |  |
|  | 9+3=8 |  |  |
|  | 9X3=32 |  |  |
|  | 9x3=4 |  |  |
|  | 9x3=8 |  |  |

Appendix 4.

|  | **Emotionally negative words** | | | | | | | **Emotionally neutral words** | | | | | | | |
| --- | --- | --- | --- | --- | --- | --- | --- | --- | --- | --- | --- | --- | --- | --- | --- |
|  | Valence rating | | | Word characteristics | | | |  | Valence rating | | | Word characteristics | | | |
| Word | Total | Female | Male | Length | Frequency (Zeno et al., 2006) | Frequency (Nelson & McEvoy, 1998) | Concreteness  (Nelson & McEvoy, 1998) | Word | Total | Female | Male | Length | Frequency (Zeno et al., 2006) | Frequency (Nelson & McEvoy, 1998) | Concreteness  (Nelson & McEvoy, 1998) |
| **Hate** | 1.79 (0.82) | 1.54 | 2.05 | 4 | 28.75 | 42 | 3.86 | **Lamp** | 3.17 (0.53) | 3.19 | 3.14 | 4 | 26.75 | 18 | 6.09 |
| **Violent** | 1.72 (0.9) | 1.55 | 1.89 | 7 | 15.75 | 33 | 2.86 | **Carpet** | 3.17 (0.58) | 3.12 | 3.21 | 6 | 14.5 | 13 | 5.68 |

Valence scale: 5=very happy; 4=somewhat happy; 3= neutral, 2=somewhat sad/scared, 1= very sad/scared

| **Emotionally positive words** | | | | | | | | **Mathematics words** | | | | | | | |
| --- | --- | --- | --- | --- | --- | --- | --- | --- | --- | --- | --- | --- | --- | --- | --- |
|  | Valence rating | | | Word Characteristics | | | |  | Valence rating | | | Word Characteristics | | | |
| Word | Total | Female | Male | Length | Frequency (Zeno et al., 2006) | Frequency (Nelson & McEvoy, 1998) | Concreteness  (Nelson & McEvoy, 1998) | **Word** | Total | Female | Male | Length | Frequency (Zeno et al., 2006) | Frequency (Nelson & McEvoy, 1998) | Concreteness  (Nelson & McEvoy, 1998) |
| **Gift** | 4.75 (0.48) | 4.84 | 4.66 | 4 | 29.75 | 33 | 5.28 | **Arithmetic** |  |  |  | 10 |  | 8 | 3.85 |
| **Circus** | 4.11 (0.82) | 4.2 | 4.02 | 6 | 17.75 | 7 | 5.31 | **Ten** |  |  |  | 3 |  | 165 | 4.72 |

Valence scale: 5=very happy; 4=somewhat happy; 3= neutral, 2=somewhat sad/scared, 1= very sad/scared
